# Modulatory Effect of Probiotic Fiber (Lactobacillus reuteri) in Residual Periodontal Pockets: A Randomized, Double-Blind, Placebo-Controlled Study

**DOI:** 10.7759/cureus.77413

**Published:** 2025-01-14

**Authors:** Dasari Vanditha, Ramesh Reddy B. V., Vijay Kumar Chava, Sukrutha Biradavolu, Sreenivas Nagarakanti, Sumanth Gunupati

**Affiliations:** 1 Department of Periodontology, Narayana Dental College and Hospital, Nellore, IND; 2 Department of Periodontology, Mamatha Institute of Dental Sciences, Hyderabad, IND

**Keywords:** chronic periodontitis, dental scaling, lactobacillus reuteri, porphyromonas gingivalis, probiotics, root planing

## Abstract

Background and aim: Periodontal disease is a chronic microbial infection characterized by inflammation and destruction of periodontal tissues. Probiotics, such as *Lactobacillus reuteri*, offer potential benefits in periodontal therapy due to their antimicrobial and anti-inflammatory properties. This study aimed to evaluate the clinical and microbiological efficacy of locally administered biodegradable fibers containing *L. reuteri *as an adjunct to scaling and root planing (SRP) in managing chronic periodontitis.

Material and methods: A single-center, double-blind, placebo-controlled, parallel-arm, randomized clinical trial was conducted with 30 patients aged 30-60 years diagnosed with chronic periodontitis were randomized into test (SRP + probiotic fiber) and control (SRP + placebo fiber) groups. Clinical parameters, including plaque index (PI), bleeding index (BI), probing pocket depth (PPD), clinical attachment level (CAL), and microbial load (*Porphyromonas gingivalis *via quantitative reverse transcription-polymerase chain reaction (qRT-PCR)) were assessed at baseline and three months post-treatment. Quantification of *P. gingivalis* was conducted using real-time quantitative PCR. The Shapiro-Wilk test assessed normality, and the Mann-Whitney U and Wilcoxon tests were used for inter- and intra-group comparisons.

Results: Both groups showed significant improvements in clinical parameters (p<0.05). The test group achieved a 45.66% reduction in PI, 57.83% reduction in BI, and significant improvements in PPD (43.06%) and CAL (43.06%). Microbial load decreased more in the test group (63.00%) than in the control group (26.08%).

Conclusions: Adjunctive use of *L. reuteri* in biodegradable fiber form improves clinical outcomes, highlighting its potential as a non-invasive adjunctive treatment for chronic periodontitis. Further studies are warranted to validate long-term efficacy.

## Introduction

Chronic periodontitis is a prevalent inflammatory condition affecting the supporting structures of the teeth, including the gingiva, periodontal ligament, cementum, and alveolar bone. This disease is characterized by progressive attachment loss and bone destruction, ultimately leading to tooth loss if untreated. The primary etiology of chronic periodontitis is the accumulation of pathogenic bacteria in dental plaque biofilms, which elicit an inflammatory response from the host. Despite advances in dental care, periodontitis remains a significant public health issue due to its high prevalence and its impact on oral health-related quality of life [1].

Non-surgical periodontal therapy (NSPT), particularly scaling and root planing (SRP), is the cornerstone of periodontitis treatment. SRP aims to remove subgingival plaque and calculus, thereby reducing the bacterial load and halting the progression of periodontal destruction [2]. However, SRP alone may not always be sufficient to achieve optimal clinical outcomes, particularly in deep periodontal pockets where complete debridement is challenging [3,4]. Consequently, adjunctive therapies have been explored to enhance the effectiveness of SRP.

Probiotics, defined as “live microorganisms that confer health benefits to the host when administered in adequate amounts”, have gained considerable attention in periodontal therapy. Probiotics can modulate the host immune response, inhibit the growth of pathogenic bacteria, and enhance the colonization of beneficial microbes in the oral cavity. Among the various probiotic strains, *Lactobacillus reuteri* has shown promising results due to its ability to produce reuterin, a broad-spectrum antimicrobial compound, and its immunomodulatory properties [4,5]. Several studies have demonstrated the beneficial effects of systemic and local administration of probiotics in managing periodontal diseases. Systemic administration, typically through dietary supplements or dairy products, can influence the oral microbiota indirectly [6]. However, the local application of probiotics directly to the periodontal pocket may offer a more targeted approach, potentially enhancing their efficacy in reducing periodontal pathogens and inflammation [7].

Biodegradable delivery systems for local administration of probiotics have been developed to ensure sustained release and prolonged therapeutic effects. Polylactic acid (PLA) is a biodegradable polymer that can be used to fabricate fibers for the local delivery of probiotics. These fibers can be inserted into periodontal pockets, where they gradually degrade, releasing the probiotics over time. This method provides a continuous supply of beneficial bacteria directly to the site of infection, potentially improving clinical outcomes in periodontitis treatment [8].

The present study was designed to evaluate the clinical and microbiological efficacy of local administration of biodegradable probiotic fiber (*L. reuteri*) as an adjunctive treatment to SRP in patients with chronic periodontitis. This randomized, placebo-controlled, double-blind clinical trial aimed to compare the effects of SRP alone versus SRP combined with local probiotic therapy on clinical parameters. Additionally, the study assessed the impact of the treatments on the subgingival microbiota, specifically focusing on the levels of *Porphyromonas gingivalis*, a key periodontal pathogen.

Previous studies have shown that the use of *L. reuteri* in periodontal therapy can lead to significant improvements in clinical and microbiological outcomes. For instance, Teughels et al. demonstrated that the adjunctive use of probiotics in periodontal therapy reduced probing pocket depth (PPD) and improved clinical attachment levels (CALs) compared to SRP alone [9]. Similarly, Vivekananda et al. reported that probiotic supplementation led to significant reductions in gingival inflammation and periodontal pathogens [10]. These findings suggest that probiotics, particularly *L. reuteri*, could be a valuable adjunctive therapy in managing chronic periodontitis.

The innovative aspect of the current study lies in the use of a biodegradable fiber delivery system for the local administration of *L. reuteri*. This approach ensures sustained release and prolonged contact of the probiotics with the periodontal tissues, potentially enhancing their therapeutic effects. The study also employs a robust clinical trial design, including randomization, blinding, and placebo control, to ensure the reliability and validity of the findings.

By investigating the clinical and microbiological efficacy of biodegradable probiotic fibers, this study aims to contribute to the growing body of evidence supporting the use of probiotics in periodontal therapy. If proven effective, this approach could offer a novel, non-invasive treatment modality for chronic periodontitis, improving patient outcomes and reducing the burden of periodontal disease.

## Materials and methods

The purpose of this research, which was carried out at the Narayana Dental College and Hospital's Department of Periodontology in Nellore, India, was to assess the microbiological and clinical effectiveness of locally administering biodegradable probiotic fiber in addition to SRP as a treatment for chronic periodontitis. The study was a single-center, split-mouth, placebo-controlled, double-blind, parallel-arm, randomized clinical trial. The trial was registered at the Clinical Trials Registry-India (CTRI/2018/01/011422).

The study was conducted following the Helsinki Declaration of 1975, revised in 2013. The protocol was reviewed and approved by the Institutional Ethical Committee (NDC/IECC/PER/10-16/64). Every eligible patient completed an informed consent form after being made aware of the nature of the study, its possible dangers, and its benefits. The study ran from February 2018 to August 2018.

Sample size calculation

The sample size was calculated using the Statistical Package for the Social Sciences (IBM SPSS Statistics for Windows, IBM Corp., Version 21, Armonk, NY). Based on data presented in a previous study [9], hypothesis testing for two means (equal variances) with a power of 85% a two-sided, effect size of 0.8, and alpha error of 5% with a reduction in the* P. gingivalis* between groups was used with a mean difference of 50.02, a total sample size of 30 patients was required in each group. To avoid an under-power sample size due to an overestimation of the expected difference between both groups, a total of 35 patients were enrolled. Potential dropouts of 10% were included in the sample size calculation.

Preparation of probiotic and placebo fibers

The materials used in this study include probiotic bacteria (*L. reuteri*); a polymer, PLA with a molecular weight of 60,000, sourced from Sigma Aldrich Pvt. Ltd.; and probiotic and placebo fibers, prepared by the Department of Pharmaceutics, Bapuji Pharmacy College, Davanagere, India. The probiotic fibers were prepared by adding 5 grams of *L. reuteri* to a 4% acetic acid solution, which was then mixed with a pH 13 aqueous solution containing 20 wt% potassium hydroxide (KOH) and 50 wt% ethylene glycol. The mixture was pressed into fiber fragments. Using a doctor blade, 3 mL of ethyl acetate was added between the probiotic fibrous meshes after dissolving 1 gram of PLA in 6 mL of methylene chloride. To eliminate any remaining solvents, the solvents were evaporated in air at 25˚C for 24 hours and then dried in a vacuum for an additional 48 hours. The biodegradable probiotic fiber starts dissolving within 24 hours after placing it into the sulcus and completes its dissolution within four days. Whereas the placebo fiber was prepared identically but without the addition of *L. reuteri.*

In vitro degradation of the membrane

One milligram of the sample membranes was placed into glass vials containing 10 mL of phosphate-buffered saline (PBS) at pH 7.4 and 20 mg/mL of lysozyme. The vials were shaken at 15 rpm while submerged in a 37˚C water bath. For a maximum of 12 weeks, samples were taken out and replaced with new medium every day. After that, they were freeze-dried, and the amount of weight loss was calculated.

Participants

Thirty patients aged 30-60 years, diagnosed with chronic periodontitis, were selected for the study based on specific inclusion and exclusion criteria. Inclusion criteria included probing depths of ≥5 mm at two bilateral or contralateral sites and a willingness to participate in the study and maintain regular appointments. Exclusion criteria encompassed the presence of systemic diseases, use of antibiotics or mouthwashes in the past six months, previous periodontal treatment within the last six months, pregnancy or lactation, and smoking.

Randomization

Subjects were assigned to either the test group (SRP plus probiotic fiber delivery) or the control group (SRP plus placebo fiber delivery) using a computer-generated randomization table. Randomization was concealed using identical containers for probiotic and placebo fibers.

Clinical procedure

**Figure 1:**
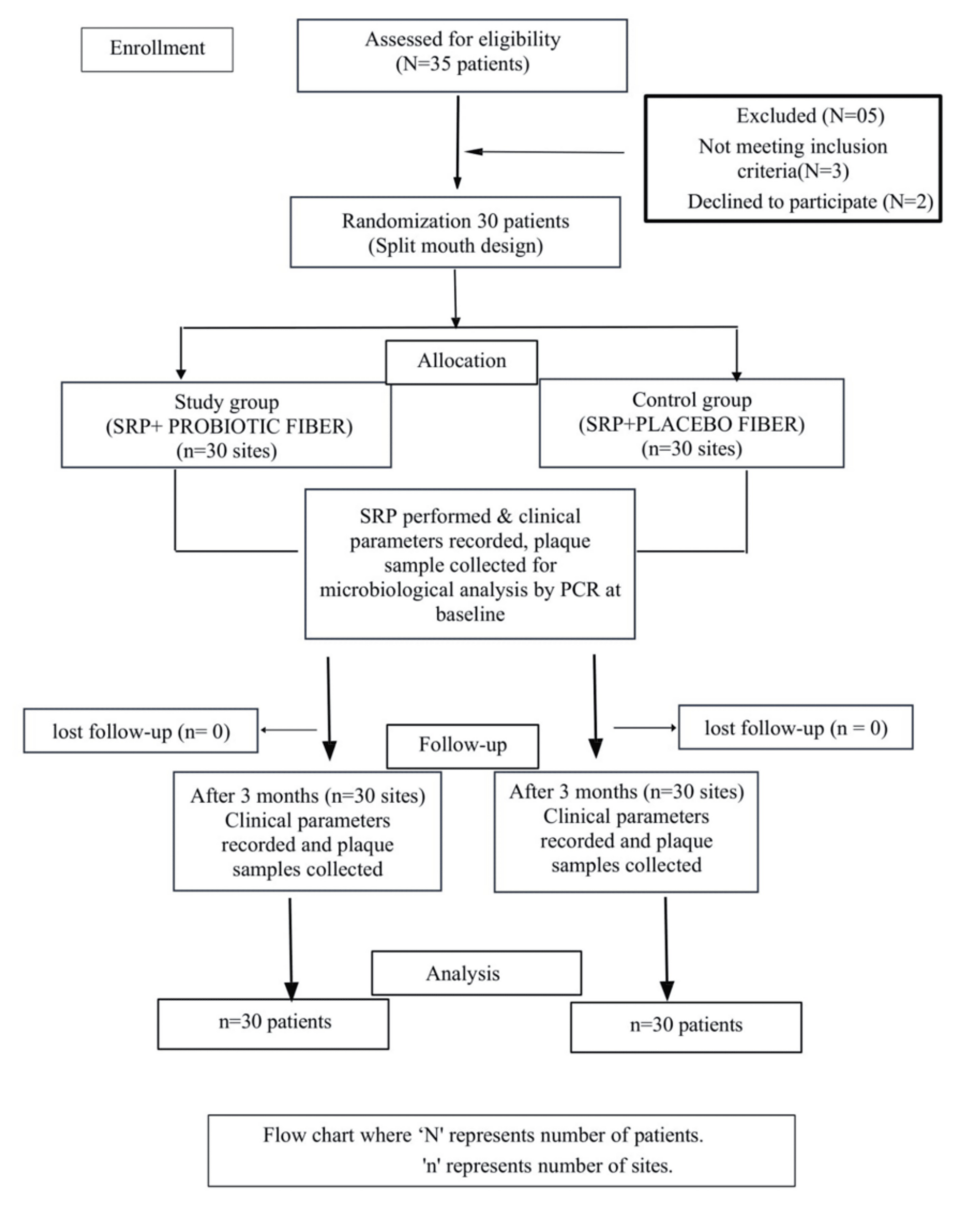
Consort flowchart for patient selection SRP: scaling and root planing; PCR: polymerase chain reaction

A split-mouth design was followed. Two sites with PPDs of ≥ 5mm at baseline were chosen from each patient, designated as S1 (site 1) and S2 (site 2), resulting in 60 sites from 30 patients, as shown in Figure 1. Subgingival plaque samples were collected using a Gracey curette.

The sites were divided into two groups: the test group: 30 sites treated with SRP followed by local probiotic fiber delivery, and the control group: 30 sites treated with SRP followed by placebo fiber delivery.

Measurement of clinical parameters

The clinical parameters measured, including plaque index (PI), sulcus bleeding index (BI), PPD, and CAL, were recorded at baseline and three months post-treatment. Probing was standardized using an acrylic stent.

Stent preparation

Impressions of upper and lower arches were taken by using alginate, and casts were poured with dental stone. Acrylic stents were fabricated with cold cure acrylic resin to fit over the occlusal third of the teeth. A groove was cut in the stent for standardized probe entry.

Local drug delivery

Following SRP, the probiotic fiber was placed in the periodontal pocket, filling the depth of the pocket, and hand pressure was applied to encourage hemostasis. After insertion, the patients were instructed to avoid disturbing the treated area.

Post-treatment instructions

Post-treatment instructions included avoiding chewing hard or sticky foods for the first few hours, refraining from flossing the treated site, and not disturbing the treated area with the tongue, fingers, or toothpicks.

Microbiological assessment

Cotton rolls are used to isolate the sample site by controlling saliva flow and a dry working field is maintained, subgingival plaque samples were collected by a sterilized Gracey curette and then placed immediately into an Eppendorf tube (Eppendorf AG, Hamburg, Germany) containing Tris-EDTA medium. All Eppendorf tubes were transferred to a -90˚C refrigerator for preservation immediately within the next 60 minutes and polymerase chain reaction (PCR) was performed to detect *P. gingivalis*.

**Figure 2:**
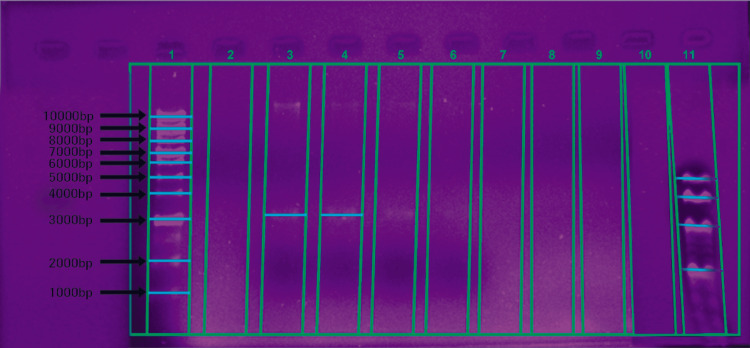
Agarose gel electrophoresis of the PCR product for the Porphyromonas gingivalis gene in chronic periodontitis patients Bands were fractionated by electrophoresis on a 2% agarose gel and visualized under UV transillumination after staining with ethidium bromide. Lanes 1, 3, 4, and 11 show positive results.

The DNA was isolated using the phenol-chloroform extraction method. The concentration and purity of DNA were measured using a NanoDrop spectrophotometer (Thermo Fisher Scientific, Waltham, USA) at 260/280 nm. The real-time quantitative PCR was used for the detection of *P. gingivalis* using specific primers targeting the 16S ribosomal RNA gene. qRT-PCR was conducted using Luna® Universal qPCR Master Mix (New England BioLabs (NEB), Ipswich, USA) and analyzed with the Bio-Rad CFX96 real-time PCR system (Bio-Rad Laboratories, Hercules, USA). The relative quantification of bacterial load was determined by comparing the results to a standard curve generated from genomic DNA. The PCR products for *P. gingivalis* were further analyzed by agarose gel electrophoresis, as shown in Figure 2.

Statistical analysis

All data were collected and entered into Microsoft Excel (Microsoft® Corp., Redmond, WA, USA) and analyzed using IBM SPSS Statistics. Descriptive statistics, including frequency, mean, and standard deviation, were calculated. A Chi-square test was used to compare gender, while the Wilcoxon matched pairs test was applied to compare control and test sites at different time intervals. The normality of scores was examined for all parameters using the Shapiro-Wilk test at baseline and at three months. The level of significance was set at < 0.05.

## Results

The clinical trial included 16 females (mean age 37.19 ± 7.39 years) and 14 males (mean age 33.93 ± 8.53 years), all of whom were systemically healthy and diagnosed with chronic periodontitis. There were no significant differences between the control and test groups at baseline, indicating that the groups were comparable at the start of the study.

The normality of scores of different parameters at baseline and three months at various treatment time points was analyzed by the Shapiro-Wilk test. The parameters did not follow the normal distribution, therefore, non-parametric tests were applied. Full mouth plaque scores were recorded at various points in time, and the results showed a substantial improvement at three-month time intervals (p<0.05) as shown in Table 1.

**Table 1:** Comparison of full mouth plaque scores at baseline and three months treatment time points * Statistically significant (p<0.05); Wilcoxon matched pairs test was used.

Time points	Mean	SD	% of change	p-value
Baseline	1.47	0.36	45.66	0.0001*
Three months	0.80	0.11		

In intergroup comparison, there was a slight difference in the scores of a BI between control and test sites at baseline to three months, but statistically, it was not significant. The reduction in PPD and gain in attachment level between control and test sites at baseline to three months had a difference in clinical values, but the improvement was not statistically significant for PPD (p=0.52) and CAL (p=0.56). The microbial analysis of the PCR scores at baseline between the control and test site was not significant statistically (p=0.21) whereas in three months the reduction was highly significant between the control and test sites (p=0.012) as shown in Table 2.

**Table 2:** Inter-group comparison of clinical and microbiological parameters of control and test sites at baseline and three months treatment time points * Statistically significant (p<0.05); Mann-Whitney U test was used. SD: standard deviation; BI: bleeding index; PPD: probing pocket depth; CAL: clinical attachment level; PCR: polymerase chain reaction

Parameter	Time points	Control site			Test site			p-value
		Mean	SD	Median	Mean	SD	Median	
BI	Baseline	2.77	0.43	3.00	2.63	0.49	3.00	0.37
	3 months	1.17	0.38	1.00	1.10	0.31	1.00	0.66
	Difference	1.60	0.62	2.00	1.53	0.68	2.00	0.78
PPD	Baseline	7.27	0.52	7.00	7.20	0.48	7.00	0.67
	3 months	3.93	1.01	3.00	4.10	1.18	4.00	0.67
	Difference	3.33	1.18	4.00	3.10	1.24	3.00	0.52
CAL	Baseline	7.27	0.45	7.00	7.20	0.41	7.00	0.66
	3 months	3.93	1.01	3.00	4.10	1.18	4.00	0.67
	Difference	3.33	1.09	4.00	3.10	1.32	3.50	0.56
log PCR	Baseline	49.17	30.67	44.11	58.75	31.97	59.38	0.21
	3 months	18.19	23.50	0.00	43.43	34.84	38.86	0.002*
	Difference	30.98	36.56	26.18	15.32	34.52	10.16	0.012*

The intragroup comparison of the change in clinical and microbiological parameters showed a statistical significance (p<0.001) in control and test sites, from baseline to three months as shown in Table 3.

**Table 3:** Intra-group comparison of all parameter scores at baseline and three months * Statistically significant (p<0.05); Wilcoxon matched pairs test was used. Z-value: standard score; BI: bleeding index; PPD: probing pocket depth; CAL: clinical attachment level; PCR: polymerase chain reaction

Parameters	Site	Mean change	% of change	Z-value	p-value
BI	Control site	1.60	57.83	4.6226	0.0001*
	Test site	1.53	58.23	4.5407	
PPD	Control site	3.33	45.87	4.7821	0.0001*
	Test site	3.10	43.06	4.7821	
CAL	Control site	3.33	45.87	4.7821	0.0001*
	Test site	3.10	43.06	4.7030	
log PCR	Control site	30.98	63.00	3.8566	0.0001*
	Test site	15.32	26.08	3.1572	0.0016*

## Discussion

The present study evaluated the clinical and microbiological efficacy of locally administered biodegradable probiotic fiber (*L. reuteri*) as an adjunct to SRP in patients with chronic periodontitis. Chronic periodontitis, characterized by inflammation and destruction of periodontal tissues, is a multifactorial disease requiring effective mechanical debridement and adjunctive therapies to enhance treatment outcomes. Probiotics, such as *L. reuteri*, have emerged as promising adjuncts due to their antimicrobial and anti-inflammatory properties. This study demonstrated significant improvements in clinical parameters and reductions in microbial load in both test and control groups, with the test group showing additional benefits in reducing plaque and inflammation.

The significant reduction in full mouth PI observed at baseline and three months is consistent with previous studies that have demonstrated the effectiveness of SRP in reducing plaque accumulation [9-12]. However, the greater reduction in PI in three months suggests that the adjunctive use of *L. reuteri *further enhances plaque control. This finding aligns with the results of Teughels et al. (2013), who reported that probiotics can effectively reduce dental plaque and improve periodontal health [9].

The BI also showed significant reductions in both groups, with the test group exhibiting more substantial improvements [10]. This outcome is indicative of reduced gingival inflammation, which is a critical component in managing periodontal diseases [13]. The anti-inflammatory properties of* L. reuteri*, as demonstrated by Krasse et al. (2006), likely contributed to the more significant reduction in BI in the test group [11]. Probiotics have been shown to modulate the host immune response, reducing the production of pro-inflammatory cytokines and promoting a healthier gingival environment [14].

PPD and CAL are critical indicators of periodontal treatment success [15]. The significant reductions in PPD and improvements in CAL observed in both groups reflect the effectiveness of SRP in managing periodontal pockets and promoting the reattachment of periodontal tissues [7,12,16-18]. The test group, which received the probiotic fiber, showed beneficial significant improvements in these parameters, suggesting that *L. reuteri* enhances the healing process. This is consistent with the findings of Vivekananda et al. (2010), who reported that probiotics can significantly improve CALs in patients with chronic periodontitis [10].

The microbiological analysis focused on the detection and quantification of *P. gingivalis*, a key periodontal pathogen. Both groups showed significant reductions in bacterial load after three months. The test group exhibited a more pronounced reduction of 30.98 (63.00%) compared to the control group, which achieved a reduction of 16.32 (26.08%). These findings highlight the efficacy of SRP in mechanically reducing bacterial load within periodontal pockets. However, the greater reduction in the test group suggests that probiotics may exert a more indirect antimicrobial effect by enhancing host immunity rather than directly eliminating pathogens [19]. *L. reuteri* is known to produce antimicrobial substances like reuterin, which can inhibit the growth of periodontal pathogens [10,14]. This aligns with the findings of Hammami and Nasri (2011) and Twetman and Jørgensen (2021), who reported that probiotic administration resulted in a significant reduction of *P. gingivalis* in periodontal pockets [11].

The significant microbial reductions reflect the complementary roles of mechanical debridement and microbial modulation in periodontal therapy. The use of real-time quantitative PCR (qRT-PCR) for detecting *P. gingivalis* provided a sensitive and specific method for detecting changes in bacterial load, further validating the results.

The Shapiro-Wilk test results revealed significant deviations from normality for most clinical parameters at baseline and three months. These deviations underscore the heterogeneity in patient responses to treatment, reflecting individual variations in disease severity, immune response, and microbial composition. The significant differences in microbial and clinical outcomes between the test and control groups highlight the complex interplay between mechanical debridement, microbial modulation, and host immune responses in periodontal therapy.

The results of this study align with findings from previous research on probiotic adjuncts in periodontal therapy. Teughels et al. (2013) and Vivekananda et al. (2010) demonstrated that *L. reuteri *significantly improved clinical and microbiological outcomes when used alongside SRP [9,10]. The novel aspect of the current study lies in the use of biodegradable fibers for local delivery of probiotics. This sustained-release system ensures prolonged contact between probiotics and periodontal tissues, enhancing their therapeutic efficacy. By contrast, systemic administration of probiotics may be less effective due to dilution and degradation during gastrointestinal transit [12]. The findings support the growing body of evidence that probiotics can complement conventional periodontal therapy by modulating microbial balance and host immunity.

The adjunctive use of *L. reuteri *in biodegradable fiber form offers a promising therapeutic approach for managing chronic periodontitis. The significant improvements in PI, BI, PPD, and gain in CAL observed in the test group highlight the potential of probiotics to enhance periodontal health outcomes. The sustained-release mechanism of the biodegradable fibers provides a practical and effective method for delivering probiotics directly to the site of infection, overcoming the limitations of systemic administration.

Probiotics represent a safe and non-invasive adjunctive therapy that aligns with the trend toward minimally invasive approaches in periodontal care. Their ability to modulate inflammation, enhance tissue healing, and promote microbial balance makes them an attractive addition to conventional treatments. The findings of this study support the integration of probiotics into clinical practice as an adjunct to SRP, particularly in patients with advanced periodontal disease or those unresponsive to mechanical therapy alone.

Study limitations and future directions

Despite its strengths, this study has certain limitations. The relatively small sample size and short follow-up period limit the generalizability of the findings. Future studies should involve larger, multi-center cohorts with longer follow-up periods to validate the results and assess the durability of clinical and microbiological improvements. Additionally, the study focused exclusively on *L. reuteri*; exploring the effects of other probiotic strains, either individually or in combination, could provide insights into optimizing probiotic formulations for periodontal therapy. The probiotic fiber is not yet commercially available as such, this study can be used for research purposes until the fiber becomes accessible on the market.

Further research is also needed to investigate the mechanisms underlying the beneficial effects of probiotics in periodontal therapy. Understanding how probiotics interact with the host immune system and influence microbial dynamics could inform the development of targeted therapies. Studies exploring the cost-effectiveness of probiotic adjuncts and their long-term impact on patient-reported outcomes would further support their clinical adoption.

## Conclusions

This study demonstrates that the local administration of biodegradable probiotic fiber (*L. reuteri*) as an adjunct to SRP significantly improves clinical and microbiological outcomes in patients with chronic periodontitis. The findings highlight the potential of probiotics to enhance plaque control, reduce inflammation, and promote tissue healing. The innovative use of biodegradable fibers ensures sustained release and prolonged therapeutic effects, offering a practical and effective method for enhancing periodontal therapy.

The results underscore the importance of integrating probiotics into periodontal care, particularly in patients with severe disease or those requiring adjunctive treatment. By modulating microbial balance and host immunity, probiotics offer a comprehensive approach to managing chronic periodontitis. Further research is warranted to explore the long-term efficacy and broader clinical applications of this promising therapeutic modality.

## References

[1] Tonetti MS, Lang NP, Cortellini P (2012). Effects of a single topical doxycycline administration adjunctive to mechanical debridement in patients with persistent/recurrent periodontitis but acceptable oral hygiene during supportive periodontal therapy. J Clin Periodontol.

[2] Jaswal R, Dhawan S, Grover V, Malhotra R (2014). Comparative evaluation of single application of 2% whole turmeric gel versus 1% chlorhexidine gel in chronic periodontitis patients: a pilot study. J Indian Soc Periodontol.

[3] Cugini MA, Haffajee AD, Smith C, Kent RL Jr, Socransky SS (2000). The effect of scaling and root planing on the clinical and microbiological parameters of periodontal diseases: 12-month results. J Clin Periodontol.

[4] Marsh PD (1992). Microbiological aspects of the chemical control of plaque and gingivitis. J Dent Res.

[5] Kõll-Klais P, Mändar R, Leibur E, Marcotte H, Hammarström L, Mikelsaar M (2005). Oral lactobacilli in chronic periodontitis and periodontal health: species composition and antimicrobial activity. Oral Microbiol Immunol.

[6] Nadelman P, Magno MB, Masterson D, da Cruz AG, Maia LC (2018). Are dairy products containing probiotics beneficial for oral health? A systematic review and meta-analysis. Clin Oral Investig.

[7] Kraft-Bodi E, Jørgensen MR, Keller MK, Kragelund C, Twetman S (2015). Effect of probiotic bacteria on oral Candida in frail elderly. J Dent Res.

[8] Jayaram P, Chatterjee A, Raghunathan V (2016). Probiotics in the treatment of periodontal disease: a systematic review. J Indian Soc Periodontol.

[9] Teughels W, Durukan A, Ozcelik O, Pauwels M, Quirynen M, Haytac MC (2013). Clinical and microbiological effects of Lactobacillus reuteri probiotics in the treatment of chronic periodontitis: a randomized placebo-controlled study. J Clin Periodontol.

[10] Vivekananda MR, Vandana KL, Bhat KG (2010). Effect of the probiotic Lactobacilli reuteri (Prodentis) in the management of periodontal disease: a preliminary randomized clinical trial. J Oral Microbiol.

[11] Krasse P, Carlsson B, Dahl C, Paulsson A, Nilsson A, Sinkiewicz G (2006). Decreased gum bleeding and reduced gingivitis by the probiotic Lactobacillus reuteri. Swed Dent J.

[12] Hammami C, Nasri W (2021). Antibiotics in the treatment of periodontitis: a systematic review of the literature. Int J Dent.

[13] Laleman I, Yilmaz E, Ozcelik O (2015). The effect of a streptococci containing probiotic in periodontal therapy: a randomized controlled trial. J Clin Periodontol.

[14] Albuquerque-Souza E, Balzarini D, Ando-Suguimoto ES, Ishikawa KH, Simionato MR, Holzhausen M, Mayer MP (2019). Probiotics alter the immune response of gingival epithelial cells challenged by Porphyromonas gingivalis. J Periodontal Res.

[15] Jones SE, Versalovic J (2009). Probiotic Lactobacillus reuteri biofilms produce antimicrobial and anti-inflammatory factors. BMC Microbiol.

[16] Twetman S, Jørgensen MR (2021). Probiotic interventions for oral health. Probiotic Research in Therapeutics.

[17] Scariya L, Nagarathna DV, Varghese M (2015). Probiotics in periodontal therapy. Int J Pharma Bio Sci.

[18] Penala S, Kalakonda B, Pathakota KR (2016). Efficacy of local use of probiotics as an adjunct to scaling and root planing in chronic periodontitis and halitosis: a randomized controlled trial. J Res Pharm Pract.

[19] Haffajee AD, Yaskell T, Torresyap G, Teles R, Socransky SS (2009). Comparison between polymerase chain reaction-based and checkerboard DNA hybridization techniques for microbial assessment of subgingival plaque samples. J Clin Periodontol.

